# QuickStats

**Published:** 2014-07-04

**Authors:** FROM THE NATIONAL CENTER FOR HEALTH STATISTICS

**Figure:**
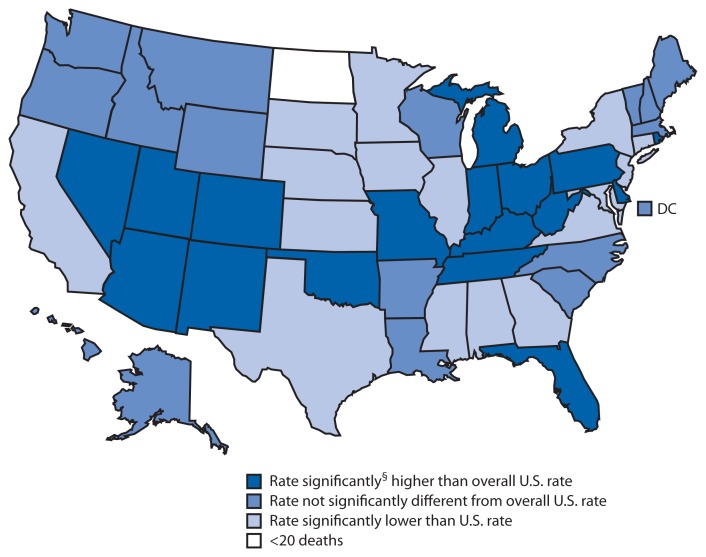
Drug-Poisoning^*^ Death Rates,^†^ by State — United States, 2011 ^*^ Based on *International Classification of Diseases, 10th Revision* codes X40–X44, X60–X64, X85, and Y10–Y14, which include deaths from all intents (unintentional, suicide, homicide, and undetermined). ^†^ Age adjusted, per 100,000 standard population. ^§^ To identify state rates that were significantly higher or lower than the overall U.S. rate of 13.2 deaths per 100,000 population, differences between the U.S. and state estimates were evaluated using two-sided significance tests at the 0.01 level.

In 2011, age-adjusted rates for deaths from drug poisoning varied by state, ranging from 7.1 to 36.3 per 100,000 population. In 17 states, the age-adjusted drug-poisoning death rate was significantly higher than the overall U.S. rate of 13.2 deaths per 100,000 population. The five states with the highest poisoning death rates were West Virginia (36.3), New Mexico (26.3), Kentucky (25.0), Nevada (22.8), and Utah (19.5).

**Sources:** National Vital Statistics System mortality data. Available at http://www.cdc.gov/nchs/deaths.htm. Death rates for drug poisoning, by state of residence, United States, 2011. Available at http://www.cdc.gov/nchs/pressroom/states/drug_deaths_2011.pdf.

**Reported by:** Li-Hui Chen, PhD, eyx5@cdc.gov, 301-458-4446; Holly Hedegaard, MD; Margaret Warner, PhD.

